# Differences in retinal microvasculature between large artery atherosclerosis and small artery disease: an optical coherence tomography angiography study

**DOI:** 10.3389/fnagi.2022.1053638

**Published:** 2022-12-23

**Authors:** Kun Lu, William Robert Kwapong, Shuai Jiang, Xuening Zhang, Jianyang Xie, Chen Ye, Yuying Yan, Le Cao, Yitian Zhao, Bo Wu

**Affiliations:** ^1^Department of Neurology, West China Hospital, Sichuan University, Chengdu, China; ^2^Cixi Institute of Biomedical Engineering, Ningbo Institute of Materials Technology and Engineering, Chinese Academy of Sciences, Ningbo, China; ^3^The Affiliated People’s Hospital of Ningbo University, Ningbo, China

**Keywords:** small artery disease, large artery atherosclerosis, retinal microvasculature, optical coherence tomography angiography, ischemic stroke

## Abstract

**Purpose**: Recent reports suggest retinal microvasculature mirror cerebral microcirculation. Using optical coherence tomography angiography (OCTA), we investigated the retinal microvasculature differences between ischemic stroke patients with large artery atherosclerosis (LAA) and small artery disease (SAD).

**Methods**: All patients underwent MR imaging and were classified as SAD and LAA; LAA was subdivided into anterior LAS and posterior LAS depending on the location. Swept-source OCTA (SS-OCTA) was used to image and segment the retina into the superficial vascular complex (SVC) and deep vascular complex (DVC) in a 6 × 6 mm area around the fovea. A deep learning algorithm was used to assess the vessel area density (VAD, %) in the retinal microvasculature.

**Results**: Fifty-eight (mean age = 60.26 ± 10.88 years; 81.03% males) were LAA while 64 (mean age = 55.58 ± 10.34 years; 85.94% males) were SAD. LAS patients had significantly reduced VAD in the DVC (*P* = 0.022) compared to SAD patients; the VAD in the SVC did not show any significant difference between the two groups (*P* = 0.580). Anterior LAA ischemic stroke showed significantly lower VAD (*P* = 0.002) in the SVC compared with posterior LAS patients. There was no significant difference in the DVC between the two groups (*P* = 0.376).

**Conclusions**: We found LAA patients had significantly reduced DVC density compared with SAD; we also showed anterior LAA patients had significantly reduced SVC density compared with posterior LAA. These findings suggest retinal imaging has the potential to be used to detect microvasculature changes in subtypes of ischemic stroke.

## Introduction

The etiologies of ischemic stroke are varied, making it difficult to include all subtypes of stroke within a single classification system (Muir, 2002). Recent advances in neuroimaging have enhanced the fitting classification and understanding of the processes of ischemic stroke, allowing therapeutic strategies to be individualized in accord with the stroke pathophysiology (Kim et al., 2013). Previous classification systems were developed in Western countries and based on Caucasians whose strokes are different from other ethnic groups.

Ischemic stroke from small artery disease (SAD) and large artery atherosclerosis (LAA) are common in the Asian populations (Kim and Kim, 2014). SAD causes one-quarter of all ischemic strokes and occurs in a single perforating artery (Jiang et al., 2020). Although neuroimaging has transformed our ability to classify the subtype of ischemic stroke, access to imaging tools is limited due to high cost, invasiveness, and lack of availability. Besides, *in vivo* visualization of cerebral microcirculation is limited due to its conspicuousness. Thus, widely available, accessible, and cost-effective screening imaging biomarkers remain a health imperative.

The brain and retina share many characteristics including embryologic origin and microvasculature. Unlike the brain, the retinal microvasculature can be visualized directly using ophthalmology techniques. Accumulating reports (Moss, 2015; Hughes et al., 2016; Cabrera DeBuc et al., 2017; Dumitrascu et al., 2018; Rim et al., 2020; Zhang J. F. et al., 2020; Biffi et al., 2022) using different ophthalmic modalities showed retinal vascular impairment is linked with cerebral vascular diseases; reports using fundus photography showed morphological changes are associated with the ischemic stroke while optical coherence tomography angiography (OCTA) showed reduced microvascular densities and fractal dimensions are linked with ischemic stroke. Using the fundus camera, Doubal et al. (2009) showed retinal vascular morphology differs between lacunar stroke and cortical stroke; the authors showed lacunar stroke patients have wider retinal venules and smaller arteriovenous ratios compared to patients with cortical stroke. Although previous reports have shown retinal microvascular changes are different in stroke subtypes, additional research is needed.

Here, we investigated the retinal microvascular changes in ischemic stroke patients with SAD compared with LAA using the swept-source OCTA (SS-OCTA). In contrast to a prior report, we provide detailed neuroimaging information and highlight the microvascular changes between these two subtypes of stroke.

## Methods

### Patients

This prospective cross-sectional study recruited acute ischemic stroke patients who were admitted to the Department of Neurology, West China Hospital, from December 2020 to March 2022. The inclusion criteria were as follows: (1) age ≥18 years old; (2) underwent magnetic resonance imaging (MRI) and OCTA imaging within 30 days of symptom onset; and (3) clinical picture suggestive of acute ischemic stroke or transient ischemic attack (focal neurologic deficits lasting <24 h).

**Figure 1:**
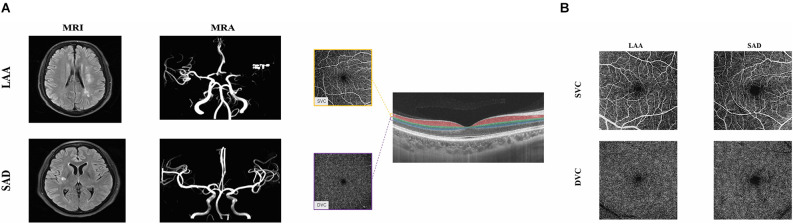
Neuroimaging and illustrative images of the retinal microvasculature network in LAA and SAD. Panel **(A)** shows the MRI and MRA images both SAD and LAA. Panel **(B)** shows the segmentation of the retinal microvasculature. En face angiograms of the superficial vascular complex (SVC) and deep vascular complex (DVC) are displayed. The right panel shows angiograms of a patient with LAA and SAD. The SVC microvascular was larger in LAA compared to SAD. LAA, large artery atherosclerosis; SAD, small artery disease.

All patients underwent a thorough stroke diagnostic workup including blood examination, carotid duplex-ultrasound, 24 h of electrocardiographic monitoring or Holter monitor, transthoracic echocardiography (TTE), and brain imaging [computer tomography (CT), MRI, and MR angiography (MRA) or CT Angiography (CTA)]. Two experienced neurologists (SJ and BW) classified the different stroke subtypes according to the modified TOAST classification (Adams et al., 1993). We only included patients who were classified as large-artery atherosclerosis (LAA) or small artery disease (SAD) as shown in Figure 1A. We excluded patients with intracranial hemorrhage, incapacity to consent, and the presence of other neurological disorders.

Patient demographics, vascular risk factors (hypertension, diabetes mellitus, dyslipidemia, coronary artery disease, atrial fibrillation, coronary heart disease, smoking, and drinking), past medical history, and neurological deficit, the National Institutes of Health Stroke Scale (NIHSS) scores at admission, were acquired systematically. Ethics approval was granted by West China Hospital Ethics Committee (2019881) and written informed consent was obtained. All procedures were done according to the Declaration of Helsinki.

### Retinal microvascular imaging

SS-OCTA (VG 200; SVision Imaging Limited, Luoyang, China) was used to scan and image the macula of all participants. The OCTA tool contained a swept-source laser with a central wavelength of 1,050 nm and a scan rate of 200,000 A-scans per second. The system was equipped with an eye-tracking utility based on an integrated confocal scanning laser ophthalmoscope to eliminate eye-motion artifacts. The axial resolution, lateral resolution, and scan depth were 5 μm, 13 μm, and 3 mm respectively.

OCTA fundus images were obtained with a raster scan protocol of 512 horizontal B-scans that covered an area of 6 × 6 mm centered on the fovea. The B-scans which contained 512 A-scans each were repeated eight times and averaged. *En face* angiograms of the superficial vascular complex (SVC) and deep vascular complex (DVC) were segmented and generated by in-built software. The two slabs were 5 μm above the inner limiting membrane (ILM) to 25 μm below the inner nuclear layer (INL). The SVC and DVC slabs were in the inner two-thirds and outer one-third of the ganglion cell layer and inner plexiform layer (GCL + IPL) as shown in Figure 1B. The quality of the macular images was assessed objectively and subjectively, rejecting images with a signal quality of less than 8 on a scale of 10. *En face* angiograms with artifacts, blurry images, and the presence of retinal diseases on images such as age-related macular degeneration (AMD), severe cataract, optic neuritis, and macular edema were also excluded.

OCTA data followed the APOSTEL recommendation (Aytulun et al., 2021).

### Quantification of the macular microvasculature based on deep learning

The OCTA-Net was utilized for microvasculature segmentation. This model consists of a split-based coarse segmentation and a split-based refining segmentation module, to produce a preliminary confidence map, and optimize the contour of the retinal microvasculature, respectively (Ma et al., 2021). The OCTA-Net was trained on a public OCTA dataset named ROSE-1, and its efficiency has been validated. Vessel area density (VAD) was calculated based on the segmentation map using MATLAB as previously reported. VAD was defined as the total length in millimeters of perfused retinal microvasculature per unit area in the analyzed area (6 × 6 mm) and is computed by applying the method proposed by Zhao and colleagues (Ma et al., 2021).

### Statistical analysis

SPSS Statistics (version 24, IBM, NY, USA) was used to perform all statistical analyses. Shapiro–Wilk test was used to assess the normality of the data. Continuous variables with normal distributions were expressed as mean ± standard deviations while skewed distributions were presented as medians and interquartile ranges. Generalized estimating equations (GEE) were used to compare the macula microvasculature between LAS and SAD patients while adjusting for risk factors (age, gender, hypertension, diabetes, dyslipidemia, smoking, and drinking) and intereye dependencies. A *P*-value less than 0.05 (*P* < 0.05) was considered statistically significant.

## Results

Of the 130 patients with ischemic stroke enrolled, eight patients did not meet our inclusion criteria. Out of the 122 ischemic stroke patients included in our data analysis, 58 (mean age = 60.26 ± 10.88 years; 81.03% males) were LAA while 64 (mean age = 55.58 ± 10.34 years; 85.94% males) were SAD. Table 1 shows the clinical information and demographics of the two groups. Noteworthy, SAD patients were younger with lower NIHSS scores compared with LAA patients as shown in Table 1.

**Table 1:** Characteristics of LAA and SAD patients.

	LAA	SAD	*P*-value
Number	58	64	
Gender, males	47	55	0.337
Age, years	60.26 ± 10.88	55.58 ± 10.34	0.016
SBP, mmHg	137.22 ± 18.37	142.22 ± 16.82	0.237
DBP, mmHg	83.59 ± 12.80	87.63 ± 18.06	0.161
Hypertension, n	39	39	0.473
Diabetes, n	18	14	0.254
Dyslipidemia, n	13	17	0.599
Smokers, n	34	30	0.144
Drinkers, n	27	22	0.124
BMI	24.69 ± 3.38	24.25 ± 4.20	0.53
NIHSS	0 (0–1)	3 (2–5)	<0.001

*SBP, systolic blood pressure; DBP, diastolic blood pressure; BMI, body mass index; NIHSS, NIH Stroke Scale; LAA, large artery atherosclerosis; SAD, small artery disease*.

Figure 1B shows angiograms of a patient with LAA and SAD; the SVC microvasculature was larger compared to the SAD patient. LAA patients had significantly reduced VAD in the DVC (*P* = 0.022, Table 2) compared to SAD patients; the VAD in the SVC did not show any significant difference between the two groups (*P* = 0.580, Table 2).

**Table 2:** Comparison of vessel area density between LAA and SAD.

	LAA	SAD	*P*-value
SVC, %	46.54 ± 3.66	46.02 ± 3.37	0.580
DVC, %	58.61 ± 2.01	59.11 ± 1.78	0.022
	Anterior LAA	Posterior LAA	*P*-value
SVC, %	46.40 ± 3.84	47.02 ± 2.92	0.002
DVC, %	58.89 ± 1.95	57.60 ± 1.94	0.376

*P-values were adjusted for age, gender, hypertension, diabetes, dyslipidemia, smoking, and drinking. SVC, superficial vascular plexus; DVC, deep vascular plexus; LAA, large artery atherosclerosis; SAD, small artery disease*.

LAA patients were stratified into the location of stroke, i.e., anterior and posterior LAA stroke; out of the 58 LAA patients, 14 had posterior LAS while 44 had anterior LAS. Patients with anterior LAS ischemic stroke showed significantly lower VAD (*P* = 0.002, Table 2) in the SVC compared with posterior LAA patients. There was no significant difference in the DVC between the two groups (*P* = 0.376, Table 2).

## Discussion

Accumulating reports (Ong et al., 2013; Kumar, 2017; Wu et al., 2017; Cao et al., 2020; Kwapong et al., 2021a, b; Liew et al., 2021) using different retinal vascular imaging tools demonstrated patients with ischemic stroke have reduced microvascular densities, sparser microvasculature, and lower microvascular fractals when compared with controls. Using the OCTA in this study, we showed retinal microvasculature differs between LAA and SAD ischemic stroke subtypes. We also showed patients with anterior LAA had significantly reduced VAD in the SVC compared with posterior LAA patients. Taken together, we suggest that there may be a dissimilar microvascular rarefaction in subtypes of ischemic stroke.

Previous reports (Ikram et al., 2006; McGeechan et al., 2009; De Silva et al., 2012; Cheung et al., 2013; Wu et al., 2017; Lemmens et al., 2020) using fundus photography showed retinal vasculopathy is associated with ischemic stroke; enlargement of venules, thinning of the arterioles, lower arteriolar/venular diameter ratio and lower fractal dimension have been linked with the incidence of ischemic stroke. Doubal et al. (2009) showed enlarged retinal venules and decreased arteriovenous ratio are linked with a lacunar stroke rather than a cortical stroke. OCTA reports have also shown patients with ischemic stroke have significantly reduced microvascular densities and lower microvascular fractal dimensions compared with controls. Using the OCTA, Zhang Y. et al. (2020) showed retinal microvascular changes did not differ between LAA and small artery occlusion lacunar stroke. Interestingly, we showed that LAA patients had significantly lower microvascular density in the DVC compared with SAD patients. Patients included in our study underwent MR imaging and OCTA imaging within 30 days of symptom onset (acute phase). The DVC is thinner and has a smaller cross-section (capillaries) making it sensitive to the disease progression, unlike the SVC which contains microvessels with a large diameter. Since the DVC is made of capillaries and is responsible for oxygen diffusion, it is susceptible to ischemia and hypoxia.

Blood supply to the brain is separated into the anterior and posterior segments, in relation to the different arteries that supply the brain. The internal carotid arteries supply the anterior brain while the vertebral arteries supply the posterior brain. Our current report showed patients with anterior LAA had significantly reduced SVC density compared with posterior LAA. Similar to the anterior cerebral circulation, the retina receives its blood supply from the internal carotid artery (Campbell et al., 2017) suggesting that changes in the anterior circulation of the brain may be reflected in the retina. From an anatomical standpoint, the retinal microvasculature reflects the cerebral microcirculation and during ischemic conditions, the SVC is suggested to be severely impaired because it is the entry of blood flow into the retina from the carotid artery (Campbell et al., 2017; Nesper and Fawzi, 2018). A previous report (Klein et al., 2000) using fundus photography speculated that the retinal vessels (SVC) are a risk indicator of ischemic stroke which may explain why the SVC was significantly reduced in anterior LAA patients compared with posterior LAA patients. On the other hand, atherosclerosis, which causes narrowing of the carotid arteries, has been suggested to be seen in the retinal arterioles and venules found in the SVC (Klein et al., 2000; Mitchell et al., 2005; Kawasaki et al., 2012; Li et al., 2022); this may also explain why the SVC impairment was sensitive in anterior LAA compared with posterior LAA.

We would like to acknowledge some limitations to our study. Firstly, we did not recruit controls in our study; we aimed to focus on the retinal microvascular changes between the two subtypes of ischemic stroke rather than the comparison between ischemic stroke and controls as previously reported. Secondly, we did not include the location of vascular lesions, and the degree of stenosis which may impact the flow of blood. Future studies with detailed information on these parameters may be needed. Retinal imaging with OCTA requires participants to focus and cooperate during imaging; thus, we included patients with little neurological deficit who could cooperate with OCTA imaging. Portable OCTA tools in the future, if possible, may provide an examination for severe cases. Low signal quality (which may occur due to media opacities) during imaging may affect the quality of retinal angiograms; thus, our study included a signal quality of eight or more in our data analysis. Head movement and severe eye movement can cause false OCTA signals/artifacts on angiograms; patients with artifacts on angiograms were excluded. Another limitation is the observational, cross-sectional design of the study which precludes inferences on the temporal link on the retinal microvasculature; longitudinal studies with larger sample sizes and detailed information on the ischemic stroke subtypes are needed.

The results of this study confirm that SAD and LAA are cerebrovascular diseases that are associated with changes in the retinal microvasculature, and the degree and level of microvascular impairment depend on the subtype of the stroke. In conclusion, we showed that LAA patients had significantly reduced DVC density compared with SAD patients suggesting a distinct microvascular change between the two ischemic stroke subtypes. Importantly, we showed anterior LAA patients had significantly reduced SVC density compared with posterior LAA patients suggesting anterior LAA may be sensitive to retina microvasculature. Taken together, we suggest retinal imaging has the potential to be used to detect microvasculature changes in subtypes of ischemic stroke.

## Data Availability Statement

The raw data supporting the conclusions of this article will be made available by the authors, without undue reservation.

## Ethics Statement

The studies involving human participants were reviewed and approved by Ethics Committee on Biomedical Research, West China Hospital of Sichuan University. The patients/participants provided their written informed consent to participate in this study.

## Author Contributions

All authors listed have made a substantial, direct, and intellectual contribution to the work and approved it for publication.
